# Supplementary material for: N-glycosylation-defective splice variants of neuropilin-1 promote metastasis by activating endosomal signals
Source: Nat Commun. 2019 Aug 16;10:3708. doi: 10.1038/s41467-019-11580-4 (PMC6697747; doi:10.1038/s41467-019-11580-4)
Supplement: Supplementary file 1 — Supplementary Information [file 41467_2019_11580_MOESM1_ESM.pdf]

Supplementary Information

N-glycosylation-defective splice variants of neuropilin-1 promote metastasis by activating endosomal signals

Huang et al.

```
NRP1_WT        MERGLPLLCAVLALVLAPAGAFRNDKCGDTIKIESPGYLTSPGYPHSYHPSEKCEWLIQA     60
NRP1_ΔE4       MERGLPLLCAVLALVLAPAGAFRNDKCGDTIKIESPGYLTSPGYPHSYHPSEKCEWLIQA     60
NRP1_ΔE5       MERGLPLLCAVLALVLAPAGAFRNDKCGDTIKIESPGYLTSPGYPHSYHPSEKCEWLIQA     60
               ************************************************************

NRP1_WT        PDPYQRIMINFNPHFDLEDRDCKYDYVEVFDGENENGHFRGKFCGKIAPPPVVSSGPFLF     120
NRP1_ΔE4       PDPYQRIMINFNPHFDLEDRDCKYDYVEVFDGENENGHFRGKFCGKIAPPPVVSSGPFLF     120
NRP1_ΔE5       PDPYQRIMINFNPHFDLEDRDCKYDYVEVFDGENENGHFRGKFCGKIAPPPVVSSGPFLF     120
               ************************************************************
                                           150
NRP1_WT        IKFVSDYETHGAGFSIRYEIFKRGPECSQNYTTPSGVIKSPGFPEKYPNSLECTYIVFAP     180
NRP1_ΔE4       IKFVSDYETHGAGFSIRYEIFKR-------------------------------------     143
NRP1_ΔE5       IKFVSDYETHGAGFSIRYEIFKRGPECSQNYTTPSGVIKSPGFPEKYPNSLECTYIVFAP     180
               ***********************

NRP1_WT        KMSEIILEFESFDLEPDSNPPGGMFCRYDRLEIWDGFPDVGPHIGRYCGQKTPGRIRSSS     240
NRP1_ΔE4       ---------------------------------------VGPHIGRYCGQKTPGRIRSSS     164
NRP1_ΔE5       KMSEIILEFESFDLEPDSNPPGGMFCRYDRLEIWDGFPD---------------------     219

                                   261                                    300
NRP1_WT        GILSMVFYTDSAIAKEGFSANYSVLQSSVSEDFKCMEALGMESGEIHSDQITASSQYSTN     300
NRP1_ΔE4       GILSMVFYTDSAIAKEGFSANYSVLQSSVSEDFKCMEALGMESGEIHSDQITASSQYSTN     224
NRP1_ΔE5       -------------------------------DFKCMEALGMESGEIHSDQITASSQYSTN     248
                                              *****************************

NRP1_WT        WSAERSRLNYPENGWTPGEDSYREWIQVDLGLLRFVTAVGTQGAISKETKKKYYVKTYKI     360
NRP1_ΔE4       WSAERSRLNYPENGWTPGEDSYREWIQVDLGLLRFVTAVGTQGAISKETKKKYYVKTYKI     284
NRP1_ΔE5       WSAERSRLNYPENGWTPGEDSYREWIQVDLGLLRFVTAVGTQGAISKETKKKYYVKTYKI     308
               ************************************************************

NRP1_WT        DVSSNGEDWITIKEGNKPVLFQGNTNPTDVVVAVFPKPLITRFVRIKPATWETGISMRFE     420
NRP1_ΔE4       DVSSNGEDWITIKEGNKPVLFQGNTNPTDVVVAVFPKPLITRFVRIKPATWETGISMRFE     344
NRP1_ΔE5       DVSSNGEDWITIKEGNKPVLFQGNTNPTDVVVAVFPKPLITRFVRIKPATWETGISMRFE     368
               ************************************************************

NRP1_WT        VYGCKITDYPCSGMLGMVSGLISDSQITSSNQGDRNWMPENIRLVTSRSGWALPPAPHSY     480
NRP1_ΔE4       VYGCKITDYPCSGMLGMVSGLISDSQITSSNQGDRNWMPENIRLVTSRSGWALPPAPHSY     404
NRP1_ΔE5       VYGCKITDYPCSGMLGMVSGLISDSQITSSNQGDRNWMPENIRLVTSRSGWALPPAPHSY     428
               ************************************************************
                                                        522
NRP1_WT        INEWLQIDLGEEKIVRGIIIQGGKHRENKVFMRKFKIGYSNNGSDWKMIMDDSKRKAKSF     540
NRP1_ΔE4       INEWLQIDLGEEKIVRGIIIQGGKHRENKVFMRKFKIGYSNNGSDWKMIMDDSKRKAKSF     464
NRP1_ΔE5       INEWLQIDLGEEKIVRGIIIQGGKHRENKVFMRKFKIGYSNNGSDWKMIMDDSKRKAKSF     488
               ************************************************************

NRP1_WT        EGNNNYDTPELRTFPALSTRFIRIYPERATHGGLGLRMELLGCEVEAPTAGPTTPNGNLV     600
NRP1_ΔE4       EGNNNYDTPELRTFPALSTRFIRIYPERATHGGLGLRMELLGCEVEAPTAGPTTPNGNLV     524
NRP1_ΔE5       EGNNNYDTPELRTFPALSTRFIRIYPERATHGGLGLRMELLGCEVEAPTAGPTTPNGNLV     548
               ************************************************************

NRP1_WT        DECDDDQANCHSGTGDDFQLTGGTTVLATEKPTVIDSTIQSEFPTYGFNCEFGWGSHKTF     660
NRP1_ΔE4       DECDDDQANCHSGTGDDFQLTGGTTVLATEKPTVIDSTIQSEFPTYGFNCEFGWGSHKTF     584
NRP1_ΔE5       DECDDDQANCHSGTGDDFQLTGGTTVLATEKPTVIDSTIQSEFPTYGFNCEFGWGSHKTF     608
               ************************************************************

NRP1_WT        CHWEHDNHVQLKWSVLTSKTGPIQDHTGDGNFIYSQADENQKGKVARLVSPVVYSQNSAH     720
NRP1_ΔE4       CHWEHDNHVQLKWSVLTSKTGPIQDHTGDGNFIYSQADENQKGKVARLVSPVVYSQNSAH     644
NRP1_ΔE5       CHWEHDNHVQLKWSVLTSKTGPIQDHTGDGNFIYSQADENQKGKVARLVSPVVYSQNSAH     668
               ************************************************************

NRP1_WT        CMTFWYHMSGSHVGTLRVKLRYQKPEEYDQLVWMAIGHQGDHWKEGRVLLHKSLKLYQVI     780
NRP1_ΔE4       CMTFWYHMSGSHVGTLRVKLRYQKPEEYDQLVWMAIGHQGDHWKEGRVLLHKSLKLYQVI     704
NRP1_ΔE5       CMTFWYHMSGSHVGTLRVKLRYQKPEEYDQLVWMAIGHQGDHWKEGRVLLHKSLKLYQVI     728
               ************************************************************

NRP1_WT        FEGEIGKGNLGGIAVDDISINNHISQEDCAKPADLDKKNPEIKIDETGSTPGYEGEGEGD     840
NRP1_ΔE4       FEGEIGKGNLGGIAVDDISINNHISQEDCAKPADLDKKNPEIKIDETGSTPGYEGEGEGD     764
NRP1_ΔE5       FEGEIGKGNLGGIAVDDISINNHISQEDCAKPADLDKKNPEIKIDETGSTPGYEGEGEGD     788
               ************************************************************
               842
NRP1_WT        KNISRKPGNVLKTLDPILITIIAMSALGVLLGAVCGVVLYCACWHNGMSERNLSALENYN     900
NRP1_ΔE4       KNISRKPGNVLKTLDPILITIIAMSALGVLLGAVCGVVLYCACWHNGMSERNLSALENYN     824
NRP1_ΔE5       KNISRKPGNVLKTLDPILITIIAMSALGVLLGAVCGVVLYCACWHNGMSERNLSALENYN     848
               ************************************************************

NRP1_WT        FELVDGVKLKKDKLNTQSTYSEA     923
NRP1_ΔE4       FELVDGVKLKKDKLNTQSTYSEA     847
NRP1_ΔE5       FELVDGVKLKKDKLNTQSTYSEA     871
               ***********************
```

Supplementary Figure 1. Alignment of NRP1-WT, NRP1-ΔE4 and NRP1-ΔE5 amino acid sequences. The character N highlighted with yellow indicates the putative N-linked glycosylation sites of NRP1.

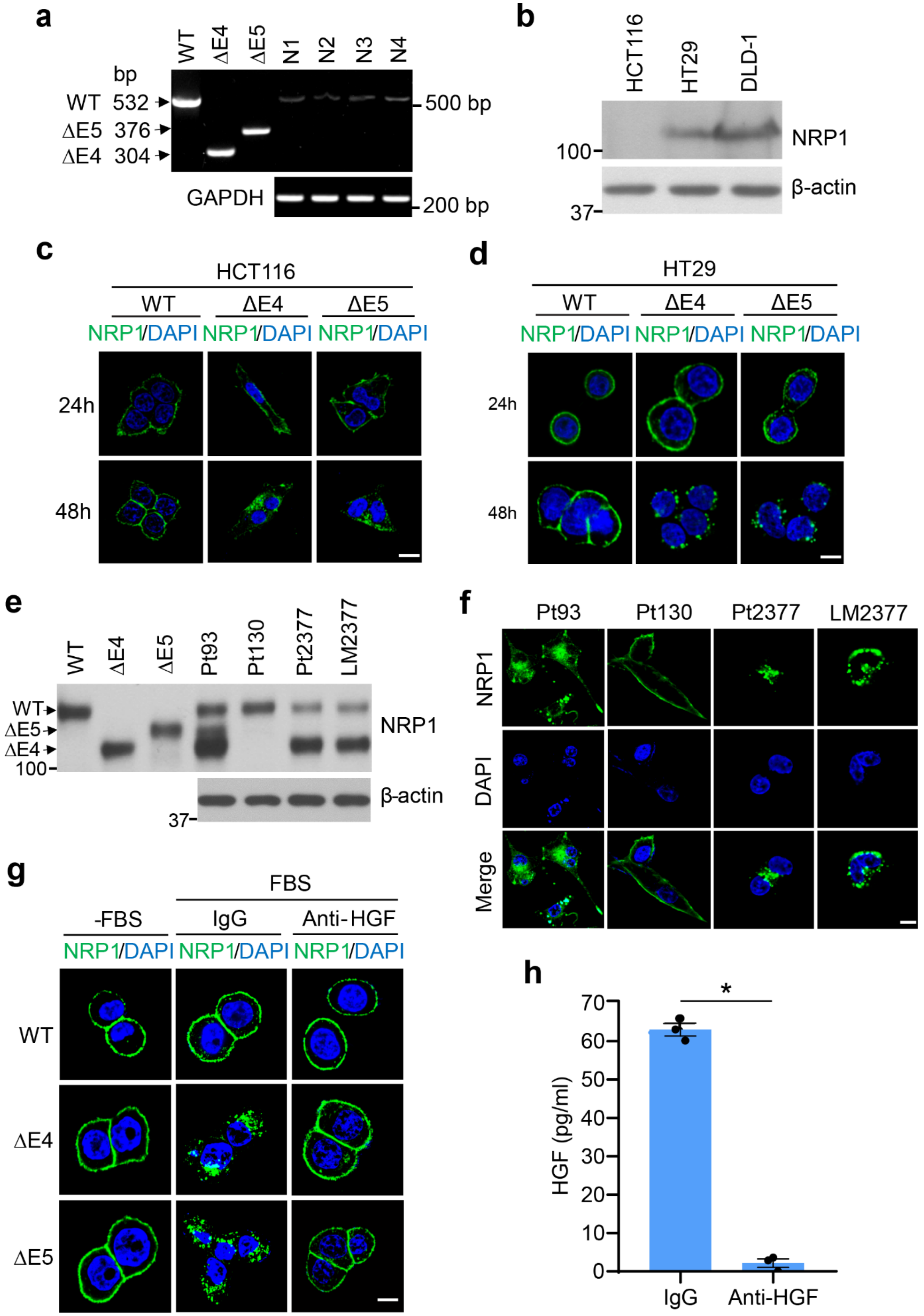
a
WT
ΔE4
ΔE5
N1
N2
N3
N4
bp
WT 532
ΔE5 376
ΔE4 304
500 bp
GAPDH
200 bp
b
HCT116
HT29
DLD-1
NRP1
β-actin
100
37
c
HCT116
WT
ΔE4
ΔE5
NRP1/DAPI
24h
48h
d
HT29
WT
ΔE4
ΔE5
NRP1/DAPI
24h
48h
e
WT
ΔE4
ΔE5
Pt93
Pt130
Pt2377
LM2377
NRP1
β-actin
100
37
f
Pt93
Pt130
Pt2377
LM2377
NRP1
DAPI
Merge
g
FBS
-FBS
IgG
Anti-HGF
NRP1/DAPI
WT
ΔE4
ΔE5
h
HGF (pg/ml)
70
60
50
40
30
20
10
0
*
IgG
Anti-HGF

Supplementary Figure 2. NRP1-∆E4 and NRP1-∆E5 are expressed in punctate cytoplasmic structures under basal conditions. (**a**) RT-PCR analysis of *NRP1*-WT mRNA expression in normal colonic mucosa was performed using the two-fold increased RNA amount than those used in Fig. 1e. (**b**) Western blot analysis of the indicated CRC cell lines. (**c, d**) Confocal images of NRP1 with DAPI staining in HCT116 (**c**) and HT29 (**d**) cells transfected with NRP1-WT, NRP1-∆E4 or NRP1-∆E5 for 24 h and 48 h. Scale bars, 10 µm. (**e**) Cell lysates were prepared from the primary CRC cell lines (Pt93, Pt130, Pt2237 and LM2237) and analyzed by western blot for the indicated proteins. The lysates of HCT116 cells expressing NRP1-WT, NRP1-ΔE5 and NRP1-ΔE4 were used as positive controls for the western blot analysis. (**f**) Confocal images of NRP1 with DAPI staining in the indicated primary CRC cells. Scale bars, 10 µm. (**g**) Confocal images of NRP1 with DAPI staining in serum-starved (-FBS) HT29 cells and the cells stimulated with HGF antibody- or control IgG-neutralized FBS for 30 min. Scale bars, 10 µm. (**h**) Quantification of HGF levels in FBS neutralized with HGF antibody or control IgG using sandwich ELISA. Data are presented as mean ± s.e.m. (n=3 independent experiments). *$p < 0.001$ using Student's t-test.

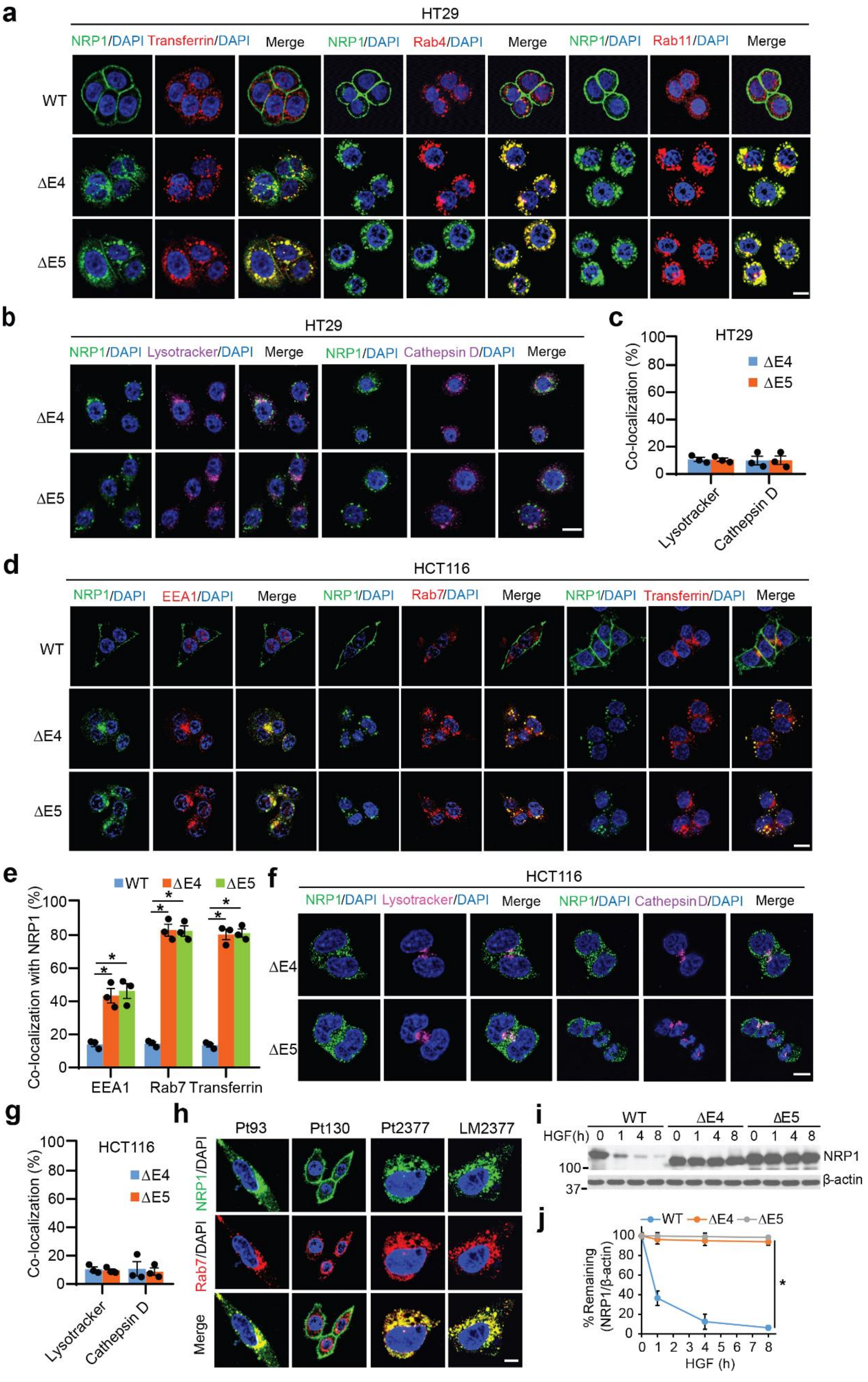

a
HT29
NRP1/DAPI
Transferrin/DAPI
Merge
NRP1/DAPI
Rab4/DAPI
Merge
NRP1/DAPI
Rab11/DAPI
Merge
WT
ΔE4
ΔE5
b
HT29
NRP1/DAPI
Lysotracker/DAPI
Merge
NRP1/DAPI
Cathepsin D/DAPI
Merge
ΔE4
ΔE5
c
HT29
Co-localization (%)
ΔE4
ΔE5
Lysotracker
Cathepsin D
d
HCT116
NRP1/DAPI
EEA1/DAPI
Merge
NRP1/DAPI
Rab7/DAPI
Merge
NRP1/DAPI
Transferrin/DAPI
Merge
WT
ΔE4
ΔE5
e
WT
ΔE4
ΔE5
Co-localization with NRP1 (%)
EEA1
Rab7
Transferrin
f
HCT116
NRP1/DAPI
Lysotracker/DAPI
Merge
NRP1/DAPI
Cathepsin D/DAPI
Merge
ΔE4
ΔE5
g
HCT116
Co-localization (%)
ΔE4
ΔE5
Lysotracker
Cathepsin D
h
Pt93
Pt130
Pt2377
LM2377
NRP1/DAPI
Rab7/DAPI
Merge
i
WT
ΔE4
ΔE5
HGF(h)
0 1 4 8
NRP1
β-actin
100
37
j
WT
ΔE4
ΔE5
% Remaining (NRP1/β-actin)
HGF (h)

Supplementary Figure 3. NRP1-∆E4 and NRP1-∆E5 co-localize with endosomal and recycling markers and exhibit a defective degradation. (**a, b**) Confocal images of NRP1, transferrin, Rab4, Rab11, lysotracker, cathepsin D and DAPI staining in HT29 cells with expression of the indicated NRP1 isoforms. Scale bars, 10 µm. (**c**) Quantification of co-localization between lysotracker or cathepsin D and the indicated NRP1 isoforms as shown in (**b**). (**d**) Confocal images of NRP1, EEA1, Rab7, transferrin and DAPI staining in HCT116 cells with expression of the indicated NRP1 isoforms. Scale bars, 10 µm. (**e**) Quantification of co-localization between the indicated NRP1 isoforms and the endosomal markers EEA1, Rab7 or transferrin as shown in (**d**). (**f**) Confocal images of NRP1, lysotracker, cathepsin D and DAPI staining in HCT116 cells with expression of the indicated NRP1 isoforms. Scale bars, 10 µm. (**g**) Quantification of co-localization between lysotracker or cathepsin D and the indicated NRP1 isoforms as shown in (**f**). (**h**) Confocal images of NRP1, Rab7 and DAPI staining in the indicated primary CRC cells. Scale bars, 10 µm. (**i**) Serum-starved HCT116 cells with expression of the indicated NRP1 isoforms were pretreated with 50 µg ml^{-1} cycloheximide and stimulated with HGF (50 ng ml^{-1}) for the indicated times followed by western blot analysis. (**j**) The western blots of NRP1 shown in (**i**) were quantified using Image J software. The level of NRP1 remaining was obtained by normalizing to the β-actin level at each time point. All graphic data are presented as mean ± s.e.m. (n=3 independent experiments). *$p < 0.003$ using Student's *t*-test.

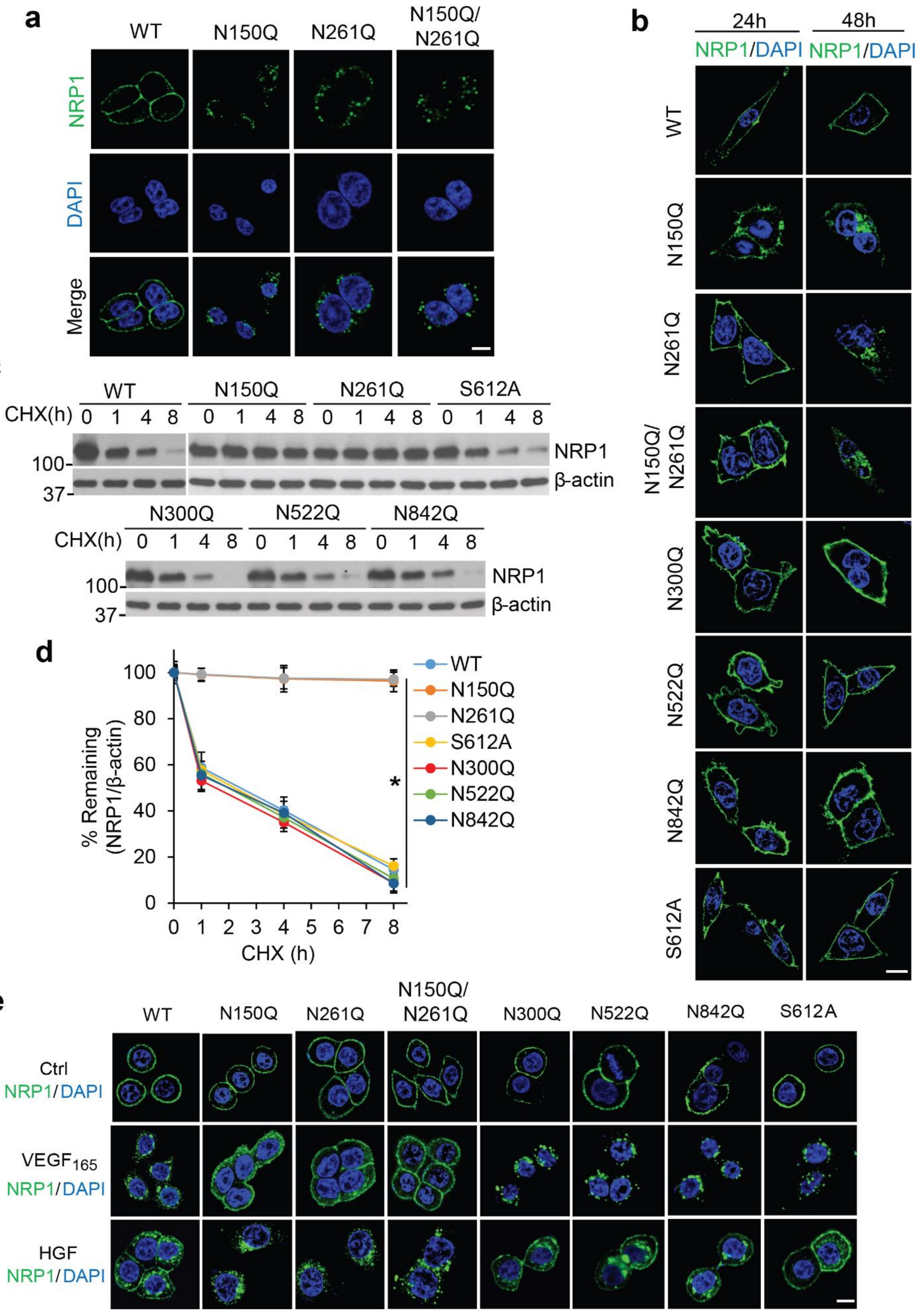

a
WT
N150Q
N261Q
N150Q/N261Q
NRP1
DAPI
Merge
b
24h
48h
NRP1/DAPI
WT
N150Q
N261Q
N150Q/N261Q
N300Q
N522Q
N842Q
S612A
c
CHX(h)
0 1 4 8
S612A
NRP1
β-actin
100
37
N300Q
N522Q
N842Q
d
% Remaining (NRP1/β-actin)
CHX (h)
*
e
Ctrl
VEGF165
HGF

Supplementary Figure 4. Defect in N150- or N261-linked glycosylation is critical for NRP1 internalization and accumulation. (**a**) Confocal images of NRP1 with DAPI staining in HT29 cells with stable expression of NRP1-WT or the indicated mutants. Scale bars, 10 μm. (**b**) Confocal images of NRP1 with DAPI staining in HCT116 cells transfected with NRP1-WT or the indicated mutants for 24 h and 48 h. Scale bars, 10 μm. (**c**) HCT116 cells with transient expression of NRP1-WT or the indicated mutants were treated with 50 μg ml^{-1} cycloheximide (CHX) for the indicated times followed by western blot analysis. (**d**) Western blots of NRP1 as shown in (**c**) were quantified using Image J software. The level of NRP1 remaining was obtained by normalizing to the β-actin level at each time point. Data are presented as mean ± s.e.m. (n=3 independent experiments). $^*p < 0.001$ using Student's *t*-test. (**e**) Confocal images of NRP1 with DAPI staining in the serum-satrved HT29 cells with expression of the indicated NRP1 isoforms, stimulated with $VEFG_{165}$ (50 ng ml^{-1}), HGF (50 ng ml^{-1}) or PBS as control for 30 min. Scale bars, 10 μm.

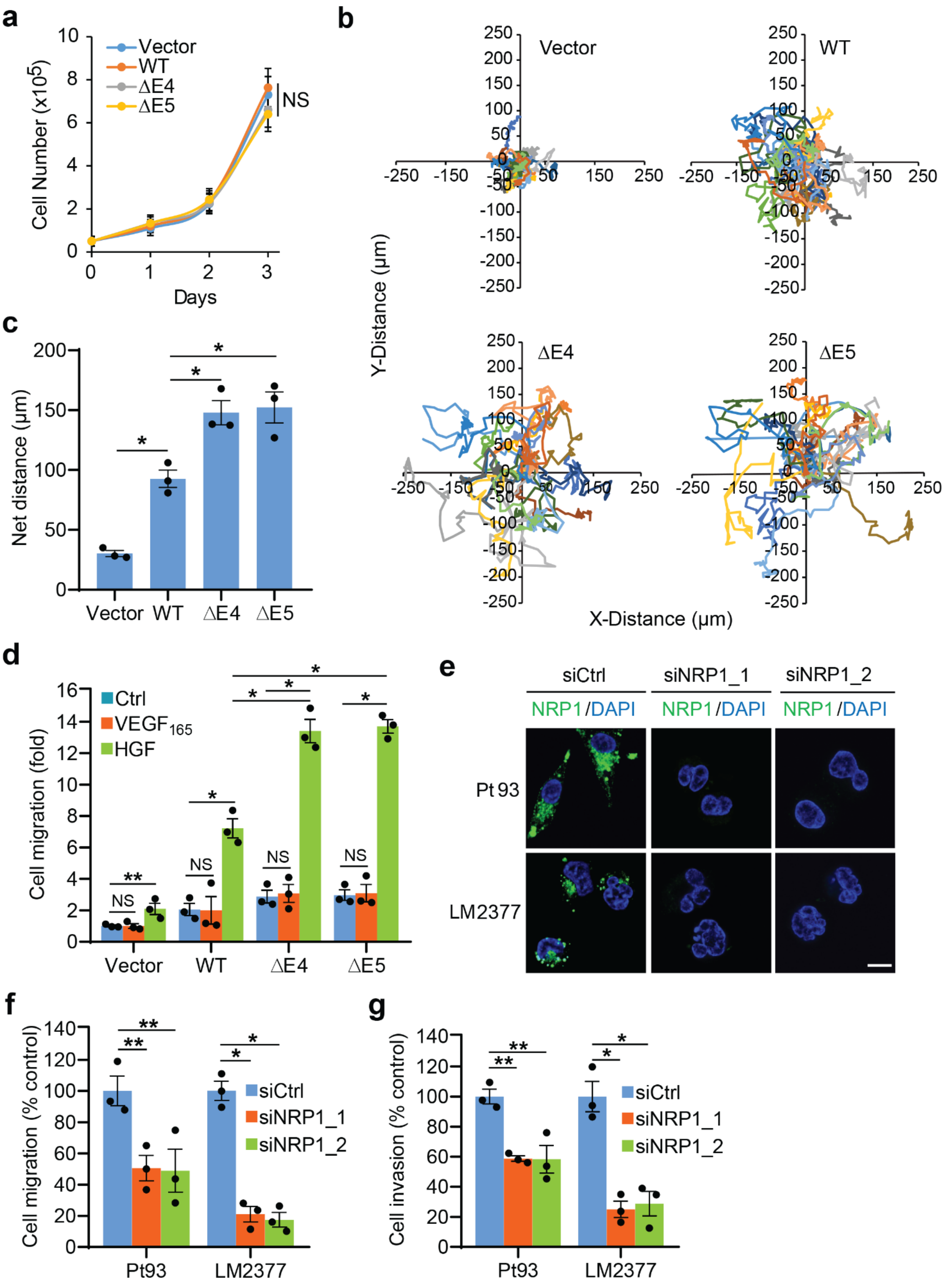

a
Cell Number (x10^5)
Days
Vector
WT
ΔE4
ΔE5
NS
b
Vector
WT
ΔE4
ΔE5
Y-Distance (μm)
X-Distance (μm)
c
Net distance (μm)
Vector
WT
ΔE4
ΔE5
d
Cell migration (fold)
Ctrl
VEGF165
HGF
NS
Vector
WT
ΔE4
ΔE5
e
siCtrl
siNRP1_1
siNRP1_2
NRP1/DAPI
Pt 93
LM2377
f
Cell migration (% control)
siCtrl
siNRP1_1
siNRP1_2
Pt93
LM2377
g
Cell invasion (% control)
siCtrl
siNRP1_1
siNRP1_2
Pt93
LM2377

Supplementary Figure 5. NRP1-ΔE4 and NRP1-ΔE5 promote CRC cell migration and invasion. (**a**) HCT116 cells with stable expression of NRP1-WT, NRP1-ΔE4, NRP1-ΔE5 or vector control were assessed for cell growth over 3 days. (**b**) Migration patterns of individual cells as determined by time-lapse imaging. The migration paths of twenty randomly chosen HCT116 cells with stable expression of NRP1-WT, NRP1-ΔE4, NRP1-ΔE5 or vector control were plotted as an x and y axis migration profile. (**c**) The net path lengths were quantified for the indicated HCT116 cells from (**b**). (**d**) Transwell migration analysis of HCT116 cells with stable expression of NRP1-WT, NRP1-ΔE4, NRP1-ΔE5 or vector control stimulated with $VEGF_{165}$ (50 ng ml^{-1}), HGF (50 ng ml^{-1}) or PBS control over 6 h of incubation. The results are expressed as the fold change of cell migration in the indicated cells relative to the vector control cells stimulated with PBS control. (**e**) Confocal images of NRP1 with DAPI staining in the primary Pt93 and LM2377 CRC cells transfected with two different sets of NRP1 siRNA or control siRNA for 48 h. Scale bars, 10 µm. (**f, g**) Pt93 and LM2377 CRC cells were transfected with two different sets of NRP1 siRNA or control siRNA for 36 h, followed by transwell migration (f) and invasion (g) analyses. The results are expressed as a percentage of migrated or invaded cells found in siCtrl cells. All graphic data are presented as mean ± s.e.m. (n=3 independent experiments). *$p < 0.01$; **$p < 0.04$; NS, not significant using Student's *t*-test.

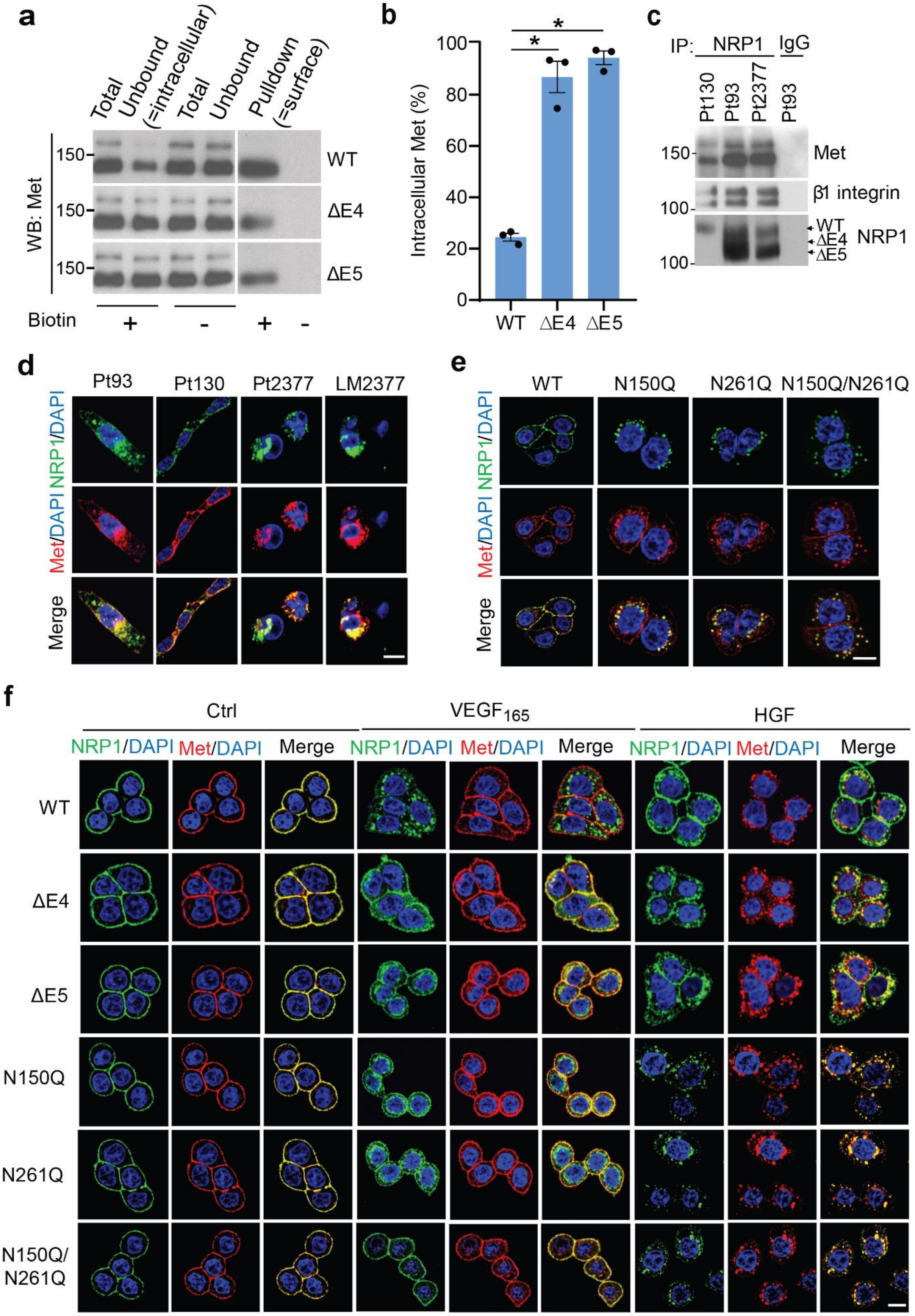
a
Total
Unbound (=intracellular)
Total
Unbound
Pulldown (=surface)
WB: Met
150
WT
ΔE4
ΔE5
Biotin
+
-
+
-
b
Intracellular Met (%)
100
80
60
40
20
0
*
*
WT
ΔE4
ΔE5
c
IP: NRP1
IgG
Pt130
Pt93
Pt2377
Pt93
150
100
Met
β1 integrin
NRP1
WT
ΔE4
ΔE5
d
Pt93
Pt130
Pt2377
LM2377
NRP1/DAPI
Met/DAPI
Merge
e
WT
N150Q
N261Q
N150Q/N261Q
f
Ctrl
VEGF165
HGF
NRP1/DAPI
Met/DAPI
Merge
WT
ΔE4
ΔE5
N150Q
N261Q
N150Q/N261Q

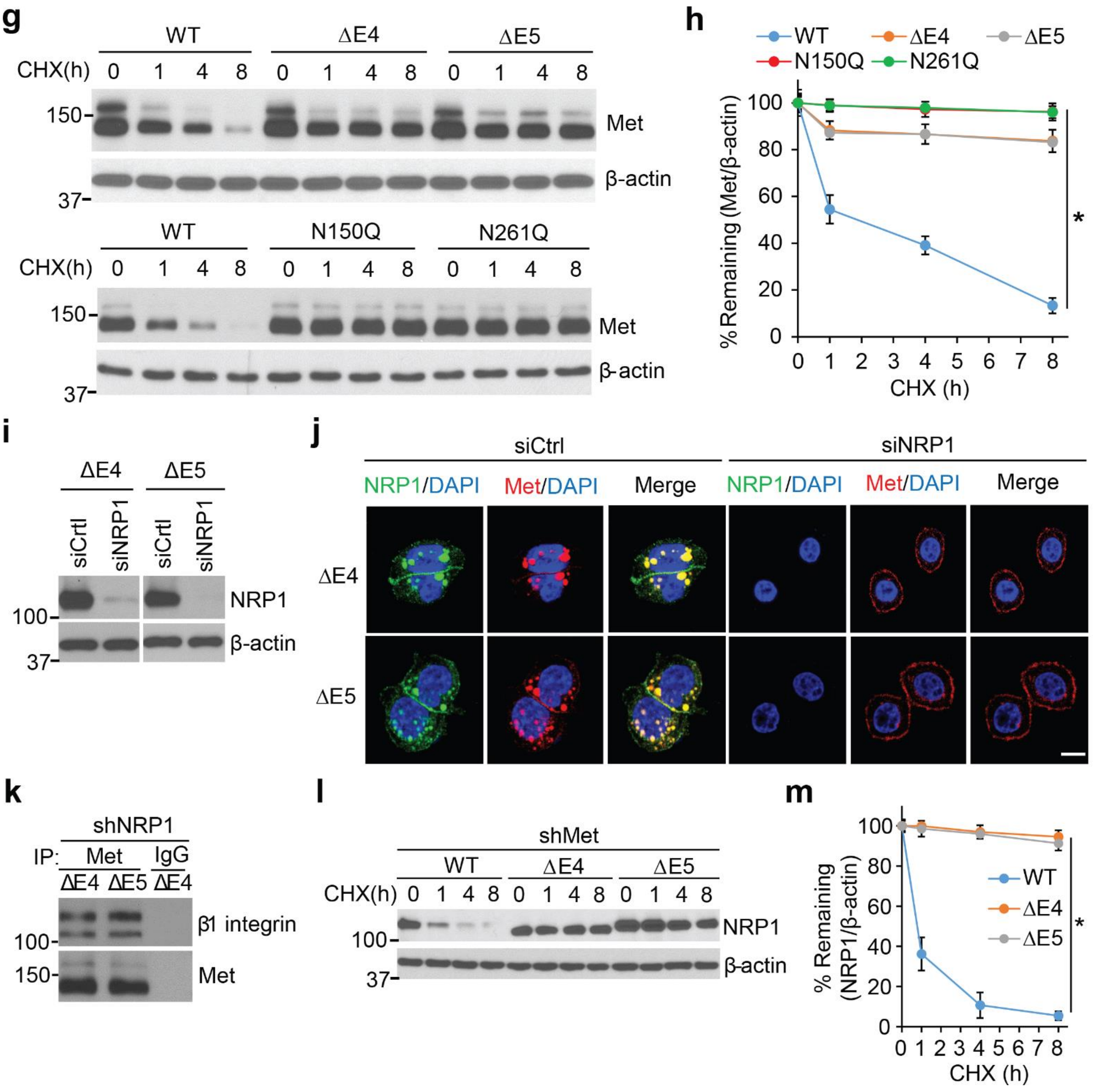

Supplementary Figure 6. N-glycosylation-defective NRP1 regulates Met internalization and stabilization. (**a**) HT29 cells with stable expression of NRP1-WT, NRP1-ΔE4 or NRP1-ΔE5 were surface-biotinylated and then the biotinylated proteins were pulled down by streptavidin beads. The total sample before pulldown (total), the supernatant corresponding to the intracellular fraction (unbound) and the surface fractions (bound) were analyzed by western blot using a Met antibody. (**b**) The percentage of intracellular Met was calculated as a ratio of the total. (**c**) The indicated primary CRC cells were lysed and immunoprecipitated with NRP1 antibody or IgG as control followed by western blot analysis. (**d, e**) Confocal images of NRP1, Met and DAPI staining in the indicated primary CRC cells (**d**) and HT29 cells with stable

expression of NRP1-WT or the indicated mutants (**e**). Scale bars, 10 µm. (**f**) Confocal images of NRP1, Met and DAPI staining in serum-satrved HT29 cells with expression of the indicated NRP1 isoforms or mutants, stimulated with $VEFG_{165}$ (50 ng ml^{-1}), HGF (50 ng ml^{-1}) or PBS as control for 30 min. Scale bars, 10 µm. (**g**) HCT116 cells with expression of the indicated NRP1 isoforms or mutants were treated with 50 µg ml^{-1} cycloheximide (CHX) for the indicated times followed by western blot analysis. (**h**) Western blots of Met as shown in (**g**) were quantified using Image J software. The level of Met remaining was obtained by normalizing to the β-actin level at each time point. Data are presented as mean ± s.e.m. (n=3 independent experiments). *$p < 0.001$ using Student's *t*-test. (**i, j**) NRP1-ΔE4- or NRP1-ΔE5-expressing HT29 cells were transfected with NRP1 siRNA or control siRNA for 48 h, followed by western blot analysis (**i**) or by confocal sections of the cells stained for NRP1 or Met with DAPI (**j**). Scale bars, 10 µm. (**k**) NRP1-ΔE4- or NRP1-ΔE5-expressing HCT116 cells with stable expression of NRP1 shRNA were lysed and immunoprecipitated with Met antibody or IgG as control followed by western blot analysis. (**l**) NRP1-WT-, NRP1-ΔE4- or NRP1-ΔE5-expressing HCT116 cells with stable expression of Met shRNA were treated with 50 µg ml^{-1} cycloheximide (CHX) for the indicated times followed by western blot analysis. (**m**) Western blots of NRP1 as shown in (**l**) were quantified using Image J software. The level of NRP1 remaining was obtained by normalizing to the β-actin level at each time point. Data are presented as mean ± s.e.m. (n=3 independent experiments). *$p < 0.001$ using Student's *t*-test.

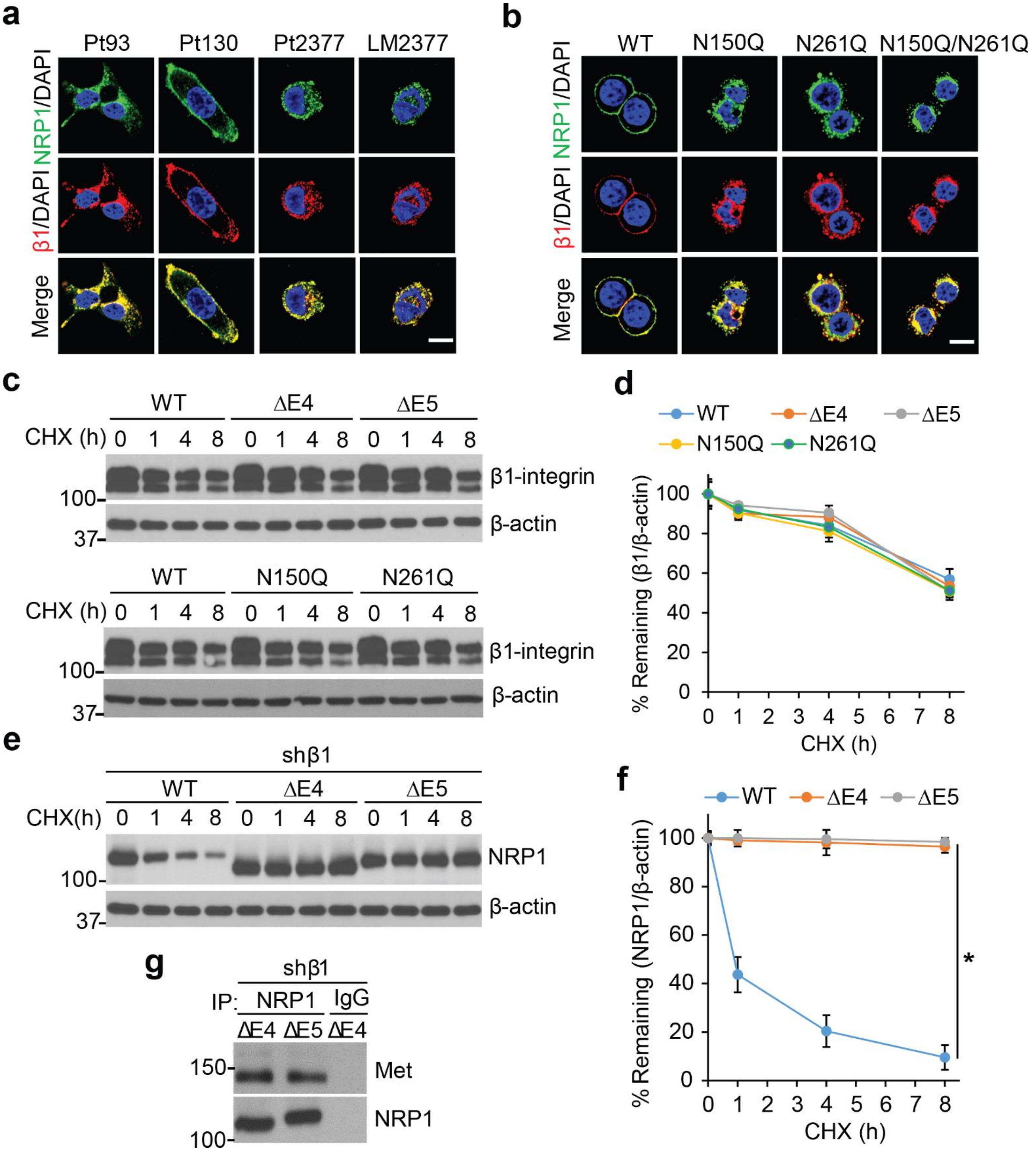

Supplementary Fig. 7. β1-integrin co-internalizes with N-glycosylation-defective NRP1. (**a**, **b**) Confocal images of NRP1, β1-integrin and DAPI staining in the indicated primary CRC cells (**a**) and HT29 cells with stable expression of NRP1-WT or the indicated mutants (**b**). Scale bars, 10 μm. (**c**) HCT116 cells with expression of the indicated NRP1 isoforms or mutants were treated with 50 μg ml^{-1} cycloheximide

(CHX) for the indicated times followed by western blot analysis. (**d**) Western blots of Met as shown in (**c**) were quantified using Image J software. The level of Met remaining was obtained by normalizing to the β-actin level at each time point. (**e**) NRP1-WT-, NRP1-ΔE4- or NRP1-ΔE5-expressing HCT116 cells with stable expression of β1-integrin shRNA were treated with 50 μg ml^{-1} cycloheximide (CHX) for the indicated times followed by western blot analysis. (**f**) Western blots of NRP1 as shown in (**e**) were quantified using Image J software. The level of NRP1 remaining was obtained by normalizing to the β-actin level at each time point. (**g**) NRP1-ΔE4- or NRP1-ΔE5-expressing HCT116 cells with stable expression of β1-integrin shRNA were lysed and immunoprecipitated with NRP1 antibody or IgG as control followed by western blot analysis. All data are presented as mean ± s.e.m. (n=3 independent experiments). *p < 0.001 using Student's t-test.

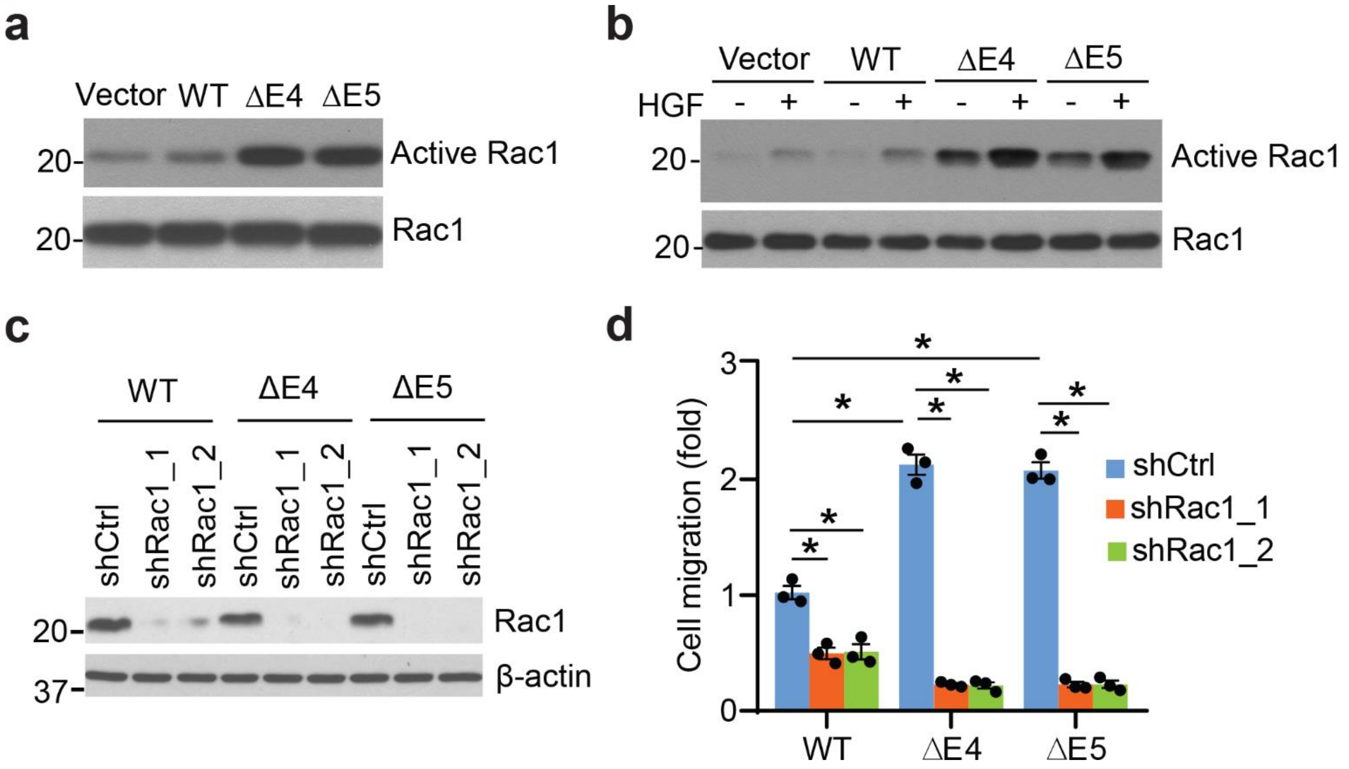

Supplementary Fig. 8. NRP1-ΔE4 and NRP1-ΔE5 increase Rac1 activity to promote CRC cell migration. (**a, b**) HCT116 cells with stable expression of the indicated NRP1 isoforms or vector control were grown in the regular growth medium containing 10% FBS (**a**), or serum starved overnight followed by stimulation with HGF (50 ng ml^{-1}) for 30 min (**b**), and subsequently assessed for the levels of Rac1-GTP and total Rac1. (**c, d**) NRP1-WT-, NRP1-ΔE4- or NRP1-ΔE5-expressing HCT116 cells with stable expression of two different sets of Rac1 shRNA or control shRNA were assessed by western blot analysis (**c**), or by transwell migration analysis (**d**). The results are expressed as the fold change over the migrated cell number found in NRP1-WT cells expressed with shCtrl. Data are presented as mean ± s.e.m. (n=3 independent experiments). *p < 0.005 using Student's t-test.

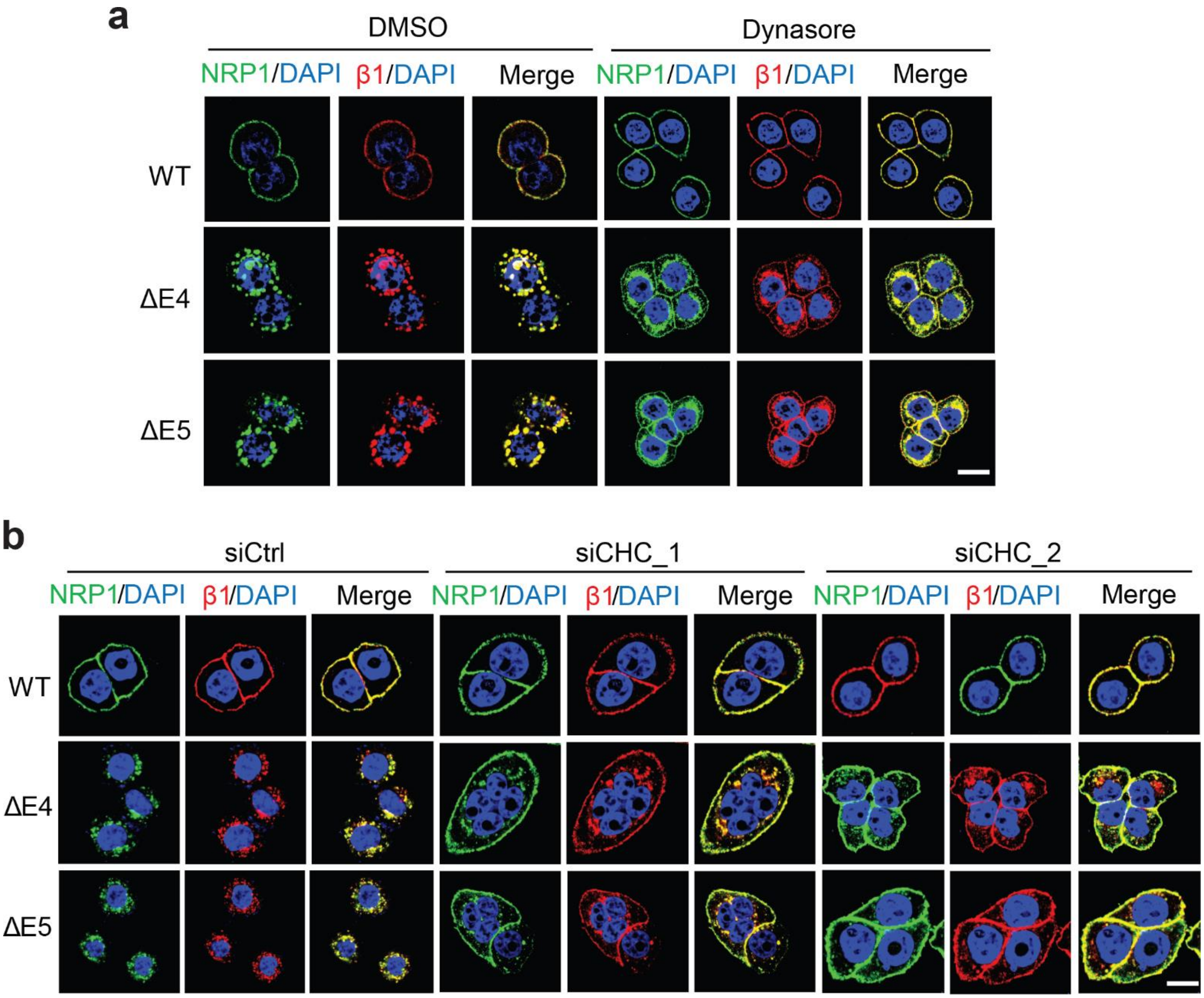

Supplementary Fig. 9. Dynasore or silencing clathrin heavy chain (CHC) reduces the co-internalization of β1-integrin with NRP1-ΔE4 or NRP1-ΔE5. (**a**) Confocal images of NRP1, β1-integrin and DAPI staining in HT29 cells with expression of the indicated NRP1 isoforms that were treated with dynasore (80 μM) or DMSO as control for 2 h. Scale bars, 10 μm. (**b**) Confocal images of NRP1, β1-integrin and DAPI staining in HT29 cells with expression of the indicated NRP1 isoforms that were transfected with two different sets of CHC siRNA or control siRNA. Scale bars, 10 μm.

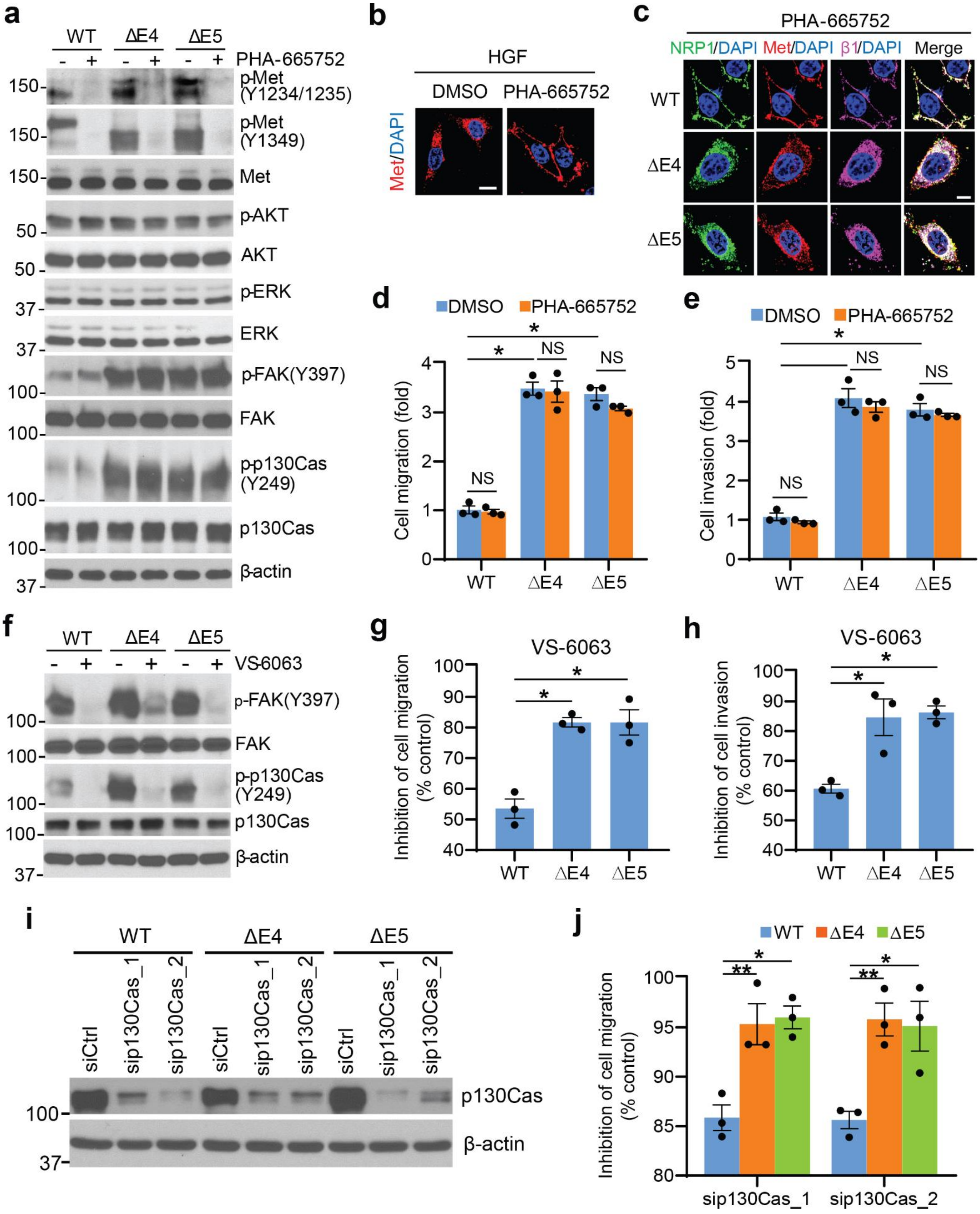
a
WT
ΔE4
ΔE5
PHA-665752
p-Met (Y1234/1235)
p-Met (Y1349)
Met
p-AKT
AKT
p-ERK
ERK
p-FAK(Y397)
FAK
p-p130Cas (Y249)
p130Cas
β-actin
b
HGF
DMSO
PHA-665752
Met/DAPI
c
PHA-665752
NRP1/DAPI
Met/DAPI
β1/DAPI
Merge
d
DMSO
PHA-665752
Cell migration (fold)
NS
e
Cell invasion (fold)
f
VS-6063
g
Inhibition of cell migration (% control)
h
Inhibition of cell invasion (% control)
i
siCtrl
sip130Cas_1
sip130Cas_2
j
sip130Cas_1
sip130Cas_2

Supplementary Figure 10. The activity of FAK kinase, but not Met tyrosine kinase, is required for NRP1-∆E4- or NRP1-∆E5-stimulated cell migration and invasion. (**a**) HCT116 cells with stable expression of NRP1-WT, NRP1-ΔE4 or NRP1-ΔE5 were treated with 1 μM PHA-665752 or DMSO as control for 6 h, followed by western blot analysis. (**b**) Confocal images of Met with DAPI staining in serum-starved HCT116 cells that were pretreated with 1 μM PHA-665752 or DMSO as control for 1 h, followed by stimulation with HGF (50 ng ml^{-1}) for 30 min. Scale bars, 10 μm. (**c**) Confocal images of NRP1, Met, β1-integrin and DAPI staining in HCT116 cells with expression of the indicated NRP1 isoforms, treated with 1 μM PHA-665752 for 1 h. Scale bars, 10 μm. (**d, e**) Transwell migration (**d**) and invasion (**e**) analyses of HCT116 cells with expression of the indicated NRP1 isoforms in the presence of DMSO or 1 μM PHA-665752 over 6 h and 30 h of incubation, respectively. The results are expressed as the fold change in cell migration or invasion in the indicated cells relative to the NRP1-WT-expressing cells treated with DMSO. (**f-h**) HCT116 cells with expression of the indicated NRP1 isoforms were treated with VS-6063 (1 μM) or DMSO for 6 h, followed by western blot analysis (**f**), or by transwell migration (**g**) and invasion (**h**) analyses. The results are expressed as the inhibition of migration or invasion relative to each of DMSO-treated control cells. (**i, j**) HCT116 cells with expression of the indicated NRP1 isoforms were transfected with two different sets of p130Cas siRNA or control siRNA for 48 h, followed by western blot analysis (**i**), or by transwell migration analysis (**j**). The results are expressed as the inhibition of migration relative to each of siCtrl cells. All graphic data are presented as mean ± s.e.m. (n=3 independent experiments). *$p < 0.01$; **$p < 0.02$ using Student's *t*-test.

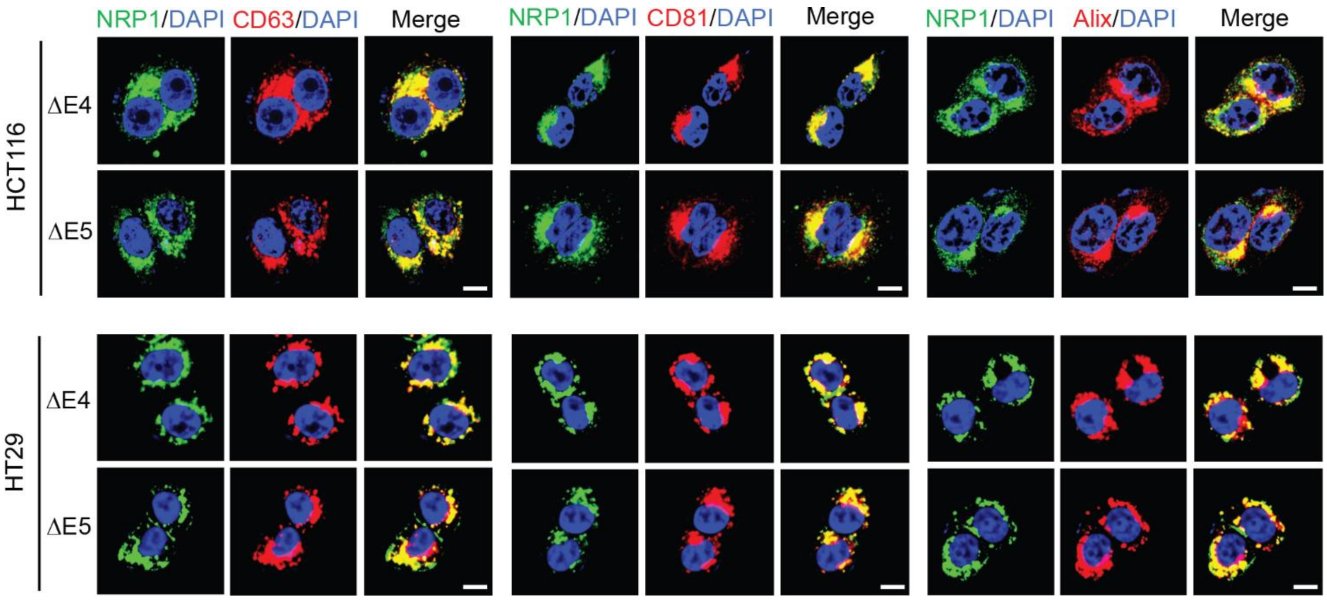

Supplementary Figure 11. NRP1-∆E4 and NRP1-∆E5 co-localize with exosome markers. Confocal images of NRP1, CD63, CD81, Alix and DAPI staining in HCT116 or HT29 cells with expression of the indicated NRP1 isoforms. Scale bars, 10 μm.

Supplementary Table 1. The use of CRC specimens in Figure 1e

Specimen	TNM Stage	Pathology	Source
Pt93	T4bN2aM1a	Invasive moderate to poorly differentiated colonic adenocarcinoma	Markey Cancer Center, University of Kentucky
Pt130	T4bN1	Metastatic colonic adenocarcinoma	Markey Cancer Center, University of Kentucky
Pt2377	T3N0M1a	Metastatic colonic adenocarcinoma	Markey Cancer Center, University of Kentucky
LM2377	T3N0M1a	Liver Metastases	Markey Cancer Center, University of Kentucky
NF90	T2N0M0	Villous colonic adenocarcinoma	Nanfang Hospital, Southern Medical University
NF99	T3N0M0	Tubule villous colonic adenocarcinoma	Nanfang Hospital, Southern Medical University
NF103	T3N0M0	Well differentiated colonic adenocarcinoma	Nanfang Hospital, Southern Medical University
NF105	T4N1aM1a	Metastatic moderate to poorly differentiated colonic adenocarcinoma	Nanfang Hospital, Southern Medical University
NF106	T3N2M0	Metastatic moderate to poorly differentiated colonic adenocarcinoma	Nanfang Hospital, Southern Medical University
NF110	T3N0M0	Moderate differentiated colonic adenocarcinoma	Nanfang Hospital, Southern Medical University

Supplementary Table 2. Clinical characteristics of patients (n = 126)

	Parameter	Number (%)
Age (yr)	<60	64
	>60	62
Sex	Male	73 (57.9%)
	Female	53 (42.1%)
Tumor location	Colon	76 (60.3%)
	Rectum	50 (39.7%)
Differentiation	Well	27 (21.4%)
	Moderate	79 (62.7%)
	Poor	20 (15.9%)
Histologic type	Adenocarcinoma	107 (84.9%)
	Mucinous carcinoma	19 (15.1%)
Stage	I	30 (23.8%)
	II	47 (37.3%)
	III	29 (23.0%)
	IV	20 (15.9%)

Supplementary Table 3. siRNA and shRNA sequences used for targeting the indicated human genes

Constructs	Target sequences (5’-3’)
NRP1 siRNA_1	AGATCGACGTTAGCTCCAA
NRP1 siRNA_2	ATCAGAGTTTCCAACATAT
Clathrin siRNA_1	AATCCAATTCGAAGACCAA
Clathrin siRNA_2	GTATGATGCTGCTAAACTA
FAK siRNA_1	CGGTCGAATGATAAGGTGT
FAK siRNA_2	CCCAGGTTTACTGAACTTA
p130Cas siRNA_1	GGTCGACAGTGGTGTGTAT
p130Cas siRNA_2	GGATGGAGGACTATGACTA
NRP1 shRNA	TGTGGATGACATTAGTATTAA
Met shRNA_1	GTGTGTTGTATGGTCAATAAC
Met shRNA_2	CCTTCAGAAGGTTGCTGAGTA
β1-integrin shRNA_1	GCCCTCCAGATGACATAGAAA
β1-integrin shRNA_2	TAGGTAGCTTTAGGGCAATAT
Rac1 shRNA_1	CGCAAACAGATGTGTTCTTAA
Rac1 shRNA_2	CGTGAAGAAGAGGAAGAGAAA
FAK shRNA	CCCAGGAGAGAATGAAGCAAA

Supplementary Table 4. Antibodies used for immunofluorescence (IF), western blot (WB) and immunoprecipitation (IP) analyses

Antibody	Usage/Dilution	Species	Provider	Catalog #
Anti-NRP1	IF: 1:100<br>IP: 1:50	Goat polyclonal	Santa Cruz Biotechnology	sc-7239
Anti-NRP1	IF: 1:100	Sheep polyclonal	R&D Systems	AF3870
Anti-NRP1	IP: 1:50	Mouse monoclonal	Santa Cruz Biotechnology	sc-5307
Anti-NRP1	WB: 1:1000	Rabbit monoclonal	Cell Signaling Technology	3725
Anti-Met	IF: 1:200	Mouse monoclonal	Cell Signaling Technology	8741
Anti-Met	IF: 1:200<br>IP: 1:50	Rabbit monoclonal	Cell Signaling Technology	8198
Anti-p-Met (Tyr1234/1235)	WB: 1:1000	Rabbit monoclonal	Cell Signaling Technology	3077
Anti-p-Met (Tyr1349)	WB: 1:1000	Rabbit monoclonal	Cell Signaling Technology	3133
Anti-EGFR	WB: 1:1000	Rabbit monoclonal	Cell Signaling Technology	4267
Anti-VEGFR2	WB: 1:1000	Rabbit monoclonal	Cell Signaling Technology	2479
Anti-AKT	WB: 1:1000	Rabbit polyclonal	Cell Signaling Technology	9272
Anti-p-Akt (Ser473)	WB: 1:1000	Rabbit monoclonal	Cell Signaling Technology	4060
Anti-ERK1/2	WB: 1:1000	Rabbit polyclonal	Cell Signaling Technology	9102
Anti-p-ERK1/2 (Thr202/Tyr204)	WB: 1:1000	Rabbit polyclonal	Cell Signaling Technology	9101
Anti-FAK	WB: 1:1000	Rabbit monoclonal	Cell Signaling Technology	13009
Anti-p-FAK (Tyr397)	WB: 1:1000	Rabbit monoclonal	Cell Signaling Technology	8556
Anti-p-FAK (Tyr397)	IF: 1:100	Rabbit polyclonal	ThermoFisher	44-624G
Anti-p130Cas	WB: 1:1000	Mouse monoclonal	Santa Cruz Biotechnology	sc-20029
Anti-p-p130Cas (Tyr249)	WB: 1:1000	Rabbit polyclonal	Cell Signaling Technology	4014
Anti-Clathrin heavy chain	WB: 1:1000	Rabbit monoclonal	Cell Signaling Technology	4796
Anti-Active β1-integrin	IF: 1:100<br>WB: 1:1000	Mouse monoclonal	Abcam	ab30394
Anti-α6-integrin	IF: 1:100	Rat monoclonal	Santa Cruz Biotechnology	sc-19622
Anti-β-actin	WB: 1:10,000	Mouse monoclonal	Sigma	A5441
Anti-EEA1	IF: 1:100	Rabbit monoclonal	Cell Signaling Technology	3288
Anti-Rab7	IF: 1:100	Rabbit monoclonal	Cell Signaling Technology	9367
Anti-Rab7	IF: 1:100	Mouse monoclonal	Cell Signaling Technology	95746
Anti-Rab4	IF: 1:50	Rabbit polyclonal	Bioss	bs-6157R
Anti-Rab11	IF: 1:100	Rabbit monoclonal	Cell Signaling Technology	5589
Anti-Cathepsin D	IF: 1:100	Mouse monoclonal	Santa Cruz Biotechnology	sc-377124
Anti-GIPC1	WB: 1:1000	Mouse monoclonal	Santa Cruz Biotechnology	sc-271822
Anti-Rac1	WB: 1:1000	Mouse monoclonal	BD Biosciences	610651
Anti-CD63	IF: 1:50	Mouse monoclonal	Santa Cruz Biotechnology	sc-5275
Anti-CD81	IF: 1:50	Mouse monoclonal	Santa Cruz Biotechnology	sc-166029
Anti-Alix	IF: 1:50	Mouse monoclonal	Santa Cruz Biotechnology	sc-53540

Anti-goat IgG with Alexa Fluor 488	IF: 1:500	Donkey polyclonal	Jackson ImmunoResearch	705-545-003
Anti-goat IgG with TRITC	IF: 1:500	Donkey polyclonal	Jackson ImmunoResearch	705-025-147
Anti-sheep IgG with Alexa Fluor 594	IF: 1:500	Donkey polyclonal	Jackson ImmunoResearch	713-585-147
Anti-rat IgG with Alexa Fluor 488	IF: 1:500	Rabbit polyclonal	Jackson ImmunoResearch	312-545-045
Anti-rabbit IgG with TRITC	IF: 1:500	Goat polyclonal	Jackson ImmunoResearch	111-025-144
Anti-mouse IgG with TRITC	IF: 1:500	Goat polyclonal	Jackson ImmunoResearch	115-025-166
Anti-rabbit IgG with Cyanine Cy5	IF: 1:500	Goat polyclonal	Jackson ImmunoResearch	111-175-144
Anti-mouse IgG with Cyanine Cy5	IF: 1:500	Goat polyclonal	Jackson ImmunoResearch	115-175-166
