# Supplementary material for: N-glycosylation-defective splice variants of neuropilin-1 promote metastasis by activating endosomal signals
Source: Nat Commun. 2019 Aug 16;10:3708. doi: 10.1038/s41467-019-11580-4 (PMC6697747; doi:10.1038/s41467-019-11580-4)
Supplement: Supplementary file 3 — Reporting Summary [file 41467_2019_11580_MOESM3_ESM.pdf]

nature research

Corresponding author(s): Side Liu, Qing-Bai She

Last updated by author(s): Jun 28, 2019

Reporting Summary

Nature Research wishes to improve the reproducibility of the work that we publish. This form provides structure for consistency and transparency in reporting. For further information on Nature Research policies, see Authors & Referees and the Editorial Policy Checklist.

Statistics

For all statistical analyses, confirm that the following items are present in the figure legend, table legend, main text, or Methods section.

n/a	Confirmed	
☐	☒	The exact sample size (*n*) for each experimental group/condition, given as a discrete number and unit of measurement
☐	☒	A statement on whether measurements were taken from distinct samples or whether the same sample was measured repeatedly
☐	☒	The statistical test(s) used AND whether they are one- or two-sided<br>*Only common tests should be described solely by name; describe more complex techniques in the Methods section.*
☐	☒	A description of all covariates tested
☒	☐	A description of any assumptions or corrections, such as tests of normality and adjustment for multiple comparisons
☐	☒	A full description of the statistical parameters including central tendency (e.g. means) or other basic estimates (e.g. regression coefficient) AND variation (e.g. standard deviation) or associated estimates of uncertainty (e.g. confidence intervals)
☐	☒	For null hypothesis testing, the test statistic (e.g. *F*, *t*, *r*) with confidence intervals, effect sizes, degrees of freedom and *P* value noted<br>*Give P values as exact values whenever suitable.*
☒	☐	For Bayesian analysis, information on the choice of priors and Markov chain Monte Carlo settings
☒	☐	For hierarchical and complex designs, identification of the appropriate level for tests and full reporting of outcomes
☒	☐	Estimates of effect sizes (e.g. Cohen's *d*, Pearson's *r*), indicating how they were calculated

Our web collection on statistics for biologists contains articles on many of the points above.

Software and code

Policy information about availability of computer code

Data collection	Nikon NIS-Elements AR software, Living Image 3.0 software, AlphaImager Mini software, Vi-Cell XR2.06.2 software and Gen5 Microplate Reader and Imager software.
Data analysis	Nikon NIS-Elements AR software, Image J software, Living Image 3.0 software, Microsoft Office Excel and GraphPad Prism (version 8). Please refer to methods section and figure legends for details.

For manuscripts utilizing custom algorithms or software that are central to the research but not yet described in published literature, software must be made available to editors/reviewers. We strongly encourage code deposition in a community repository (e.g. GitHub). See the Nature Research guidelines for submitting code & software for further information.

Data

Policy information about availability of data

All manuscripts must include a data availability statement. This statement should provide the following information, where applicable:

- Accession codes, unique identifiers, or web links for publicly available datasets
- A list of figures that have associated raw data
- A description of any restrictions on data availability

All data supporting the findings of this study are available from the corresponding authors on reasonable request. Raw data for each figure has been provided as a Source Data file.

Field-specific reporting

Please select the one below that is the best fit for your research. If you are not sure, read the appropriate sections before making your selection.

☒ Life sciences ☐ Behavioural & social sciences ☐ Ecological, evolutionary & environmental sciences

For a reference copy of the document with all sections, see nature.com/documents/nr-reporting-summary-flat.pdf

Life sciences study design

All studies must disclose on these points even when the disclosure is negative.

Sample size	For in vitro study, all the experiments were repeated at least three independent times. For animal study and analysis of human colorectal cancer tissues, samples sizes were determined based on our previous publications with the models utilized or the numbers reported in the field for statistical power.
Data exclusions	No data were excluded from the analyses.
Replication	For in vitro study, at least 3 independent experiments were performed, and all findings were reliably reproduced. For the lung metastasis experiments, 6 biologically independent mice in each group were used.
Randomization	For in vitro study, cells for different groups were randomized to be examined. The mice were randomized to put into separate groups /cages for experiments. Patients colorectal cancer samples were randomly allocated into different groups for RT-PCR analysis of the NRP1 splice variants expression.
Blinding	Experiments were performed in a blinded fashion such that the people conducting and/or analyzing the assay were not aware of treatment groups until data gathering were complete.

Reporting for specific materials, systems and methods

We require information from authors about some types of materials, experimental systems and methods used in many studies. Here, indicate whether each material, system or method listed is relevant to your study. If you are not sure if a list item applies to your research, read the appropriate section before selecting a response.

Materials & experimental systems

n/a	Involved in the study
☐	☒ Antibodies
☐	☒ Eukaryotic cell lines
☒	☐ Palaeontology
☐	☒ Animals and other organisms
☒	☐ Human research participants
☒	☐ Clinical data

Methods

n/a	Involved in the study
☒	☐ ChIP-seq
☒	☐ Flow cytometry
☒	☐ MRI-based neuroimaging

Antibodies

Antibodies used	All the antibodies used in this study have been described in detail in Supplementary Table 4.
Validation	All commercially available antibodies were validated by the suppliers. Please refer to the manufacturers' websites for details.

Eukaryotic cell lines

Policy information about cell lines

Cell line source(s)	Sources of all the cell lines used have been described in the methods section.
Authentication	All cell lines were authenticated using the short tandem repeat (STR) profiling (Genetica).
Mycoplasma contamination	All cell lines were periodically tested for mycoplasma contamination using e-Myco Plus kit (iNtRON Biotechnology). None of the cell lines were contaminated.
Commonly misidentified lines (See ICLAC register)	No commonly misidentified cell lines were used.

Animals and other organisms

Policy information about studies involving animals; ARRIVE guidelines recommended for reporting animal research

Laboratory animals	NCr Nude mice (Taconic), males, 6 weeks old
Wild animals	The study did not involve wild animals.
Field-collected samples	The study did not involve samples collected from the field.
Ethics oversight	All studies involving animals were performed under a protocol approved by the University of Kentucky Institutional Animal Care and Use Committee.

Note that full information on the approval of the study protocol must also be provided in the manuscript.
